# Acute coronary syndrome by two different spontaneous coronary artery dissection types in two different vessels

**DOI:** 10.1007/s12928-021-00783-6

**Published:** 2021-05-11

**Authors:** Youssef Salah Abdelwahed, Lukas Zanders, Ulf Landmesser, David Manuel Leistner

**Affiliations:** 1 Department of Cardiology, Charité—Universitätsmedizin Berlin, Campus Benjamin Franklin, Hindenburgdamm 30, 12203 Berlin, Germany; 2 DZHK (German Centre for Cardiovascular Research), Partner site Berlin, Berlin, Germany; 3 Berlin Institute of Health (BIH), Berlin, Deutschland

A 48-year-old, otherwise healthy woman, with a new onset recurring chest pain over a period of 1 week was admitted for coronary angiography. Surprisingly, a tubular-shaped high-grade stenosis in the middle segment of the LAD (Fig. 1a and Video 1) was revealed, simultaneous with a clear dissection seen as a radiolucent lumen resembling a Type 1 spontaneous artery dissection (SCAD), extending from the middle part of the RCA to its distal segment (Fig. 2a). Filling of the distal part of the RCA through contralateral LAD collaterals as well as TIMI II flow at LAD, led to primary imaging-guided PCI of the LAD. Optical coherence tomography imaging (OCT) showed a dissection flap and concomitant intramural hematoma (Fig. 1b) attributing to Type 3 SCAD, which was treated by primary drug-eluting-stent implantation (Fig. 1c). During a follow-up appointment after 6 weeks, the patient still reported about recurring chest pain since the first intervention. Thus, staged PCI of the RCA was performed: the proximal dissected segment was crossed into the side branch using microcatheter support, followed by exchange to a double-lumen microcatheter, allowing the safe crossing to the distal RCA lumen by a Gaia-1 wire (after failed crossing using a floppy and then a Fielder XT-A wires) (Fig. 2b and Video 2). Long-segment drug-eluting-stent PCI was performed covering the dissected part completely (Fig. 2c).

**Fig. 1:**
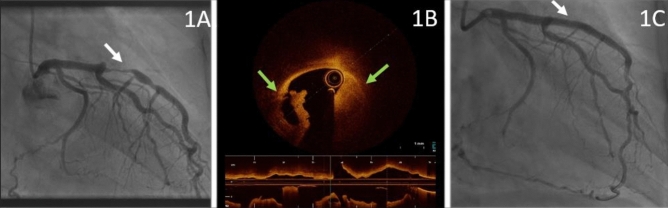
**a** Tubular high-grade stenosis in the middle part of the LAD (White Arrow). **b** OCT revealed dissection flap and intramural hematoma (Green Arrows) according SCAD Type 3, which was covered by consecutive DES-PCI (white arrow; **c**)

**Fig. 2:**
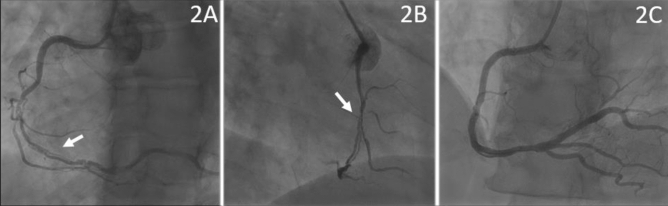
**a** RCA with radiolucent dissected lumen starting from its middle part (white arrow) according SCAD Type 1. **b** Crossing point of the wire into the true lumen of the RCA over the side branch using double-lumen microcatheter support (white arrow) with subsequent successful DES-PCI (**c**)

SCAD is now known to be an important cause of myocardial infarction in young patients [1]. Although SCAD still considered as uncommon, awareness of both the disease as well as the definition of its pathophysiologic mechanisms were approved by intracoronary high-resolution OCT imaging recently (1, 2). Here, we present a rare case, in which simultaneously two different forms—representing both rare subtypes of SCAD—could be detected and successfully treated in two different vessels in the same patient.

## Supplementary Information

Below is the link to the electronic supplementary material.

Supplementary file1 (V1) Angio, showing LAD Type 3 SCAD upon presentation (V2) RCA Type 1 SCAD and successful wire cross (AVI 24383 KB)
